# APPROACHES TO QUANTITATIVELY EXAMINE RESILIENCE IN OLD AGE

**DOI:** 10.1093/geroni/igad104.1825

**Published:** 2023-12-21

**Authors:** Rene Melis, Almar Kok, Martijn Huisman

**Affiliations:** Radboud University Medical Center, Nijmegen, Gelderland, Netherlands; Amsterdam University Medical Center, Amsterdam, Noord-Holland, Netherlands; Amsterdam University Medical Center, Amsterdam, Noord-Holland, Netherlands

## Abstract

Resilience is a complex system’s ability to maintain or recover in function following a perturbation. The concept is generic and can be applied to any complex system going from a cell, a person, to their support networks. By applying a resilience approach, we can better understand why people respond differently to a similar exposure. While most people have an intuitive understanding of what resilience is, it’s definition and application is not straightforward. Therefore, it is important to carefully define the stressor, system and (functional) outcome(s) and how it is studied using e.g., the TransNIH resilience framework. Quantitative examination of resilience can focus on predictors, mechanisms, trajectories and/or outcomes and each aspect requires a different analytical approach. This talk will review different quantitative approaches to examine resilience in the context of aging. These approaches can be categorized based on the statistical methods that are used to operationalize resilience: the effect modification approach to study how hypothesized resilience factors modify the outcome following perturbation, scale construction in the psychometric approach, comparison of characteristics between groups based on predefined prospective resilience responses in the a priori approach, data-driven subgroup identification based on resilience outcome (trajectories) in the clustering approach, analyzing predictors of adversity-outcome residual values in the residual approach, and analyzing stressor-response patterns in intensive longitudinal data to better understand resilience mechanisms. The approaches are not mutually exclusive. Researchers may choose to combine multiple approaches and may analyze the same data using multiple approaches to compare the findings between them.

